# Ste12/Fab1 phosphatidylinositol-3-phosphate 5-kinase is required for nitrogen-regulated mitotic commitment and cell size control

**DOI:** 10.1371/journal.pone.0172740

**Published:** 2017-03-08

**Authors:** David Cobley, Lenka Hálová, Marie Schauries, Adrian Kaczmarek, Mirita Franz-Wachtel, Wei Du, Karsten Krug, Boris Maček, Janni Petersen

**Affiliations:** 1 Faculty of Life Sciences, University of Manchester, Oxford Road, Manchester, M13 9PT, United Kingdom; 2 Flinders Centre for Innovation in Cancer, School of Medicine, Flinders University, Adelaide, SA, Australia; 3 South Australia Health and Medical Research Institute, North Terrace, Adelaide SA Australia; 4 Proteome Center Tübingen, Auf der Morgenstelle, Tuebingen, Germany; University of Cambridge, UNITED KINGDOM

## Abstract

Tight coupling of cell growth and cell cycle progression enable cells to adjust their rate of division, and therefore size, to the demands of proliferation in varying nutritional environments. Nutrient stress promotes inhibition of Target Of Rapamycin Complex 1 (TORC1) activity. In fission yeast, reduced TORC1 activity advances mitotic onset and switches growth to a sustained proliferation at reduced cell size. A screen for mutants, that failed to advance mitosis upon nitrogen stress, identified a mutant in the PIKFYVE 1-phosphatidylinositol-3-phosphate 5-kinase fission yeast homolog Ste12. Ste12^PIKFYVE^ deficient mutants were unable to advance the cell cycle to reduce cell size after a nitrogen downshift to poor nitrogen (proline) growth conditions. While it is well established that PI(3,5)P_2_ signalling is required for autophagy and that Ste12^PIKFYVE^ mutants have enlarged vacuoles (yeast lysosomes), neither a block to autophagy or mutants that independently have enlarged vacuoles had any impact upon nitrogen control of mitotic commitment. The addition of rapamycin to Ste12^PIKFYVE^ deficient mutants reduced cell size at division to suggest that Ste12^PIKFYVE^ possibly functions upstream of TORC1. *ste12* mutants display increased Torin1 (TOR inhibitor) sensitivity. However, no major impact on TORC1 or TORC2 activity was observed in the *ste12* deficient mutants. In summary, Ste12^PIKFYVE^ is required for nitrogen-stress mediated advancement of mitosis to reduce cell size at division.

## Introduction

In the presence of rich nutrients, cells maintain high levels of macromolecular synthesis to promote growth and increase size. Conversely, limitations in nutritional environment restrain protein synthesis to conserve crucial metabolites and promote cell division to reduce size. Thus, cells constantly monitor nutrient availability and adjust cell growth and proliferation accordingly [1, 2]. The target of rapamycin (TOR) signalling pathway is integral to this coupling. Mammalian systems possess a single TOR kinase, mTOR, whereas budding and fission yeasts contain two, Tor1 and Tor2. TOR kinase can be incorporated into two complexes, TOR complex 1 (TORC1), with Raptor as the core subunit, and TOR complex 2 (TORC2), defined by Rictor. In fission yeast, Tor1 was shown to be predominantly part of TORC2, and Tor2 was shown to be part of TORC1 [3–5].

In *S*. *pombe*, cell size control, after a shift from rich to poor nitrogen source (nitrogen stress), is dependent on TORC1 [1, 6–8]. The active TORC1 promotes cell growth to delay mitosis and cell division when good quality nutrients are present, whereas TORC1 activity decreases in response to a decline in nutrient quality and cells advance commitment to mitosis. Cells then continue to proliferate at a reduced cell size in poor nutrient environments. Inhibition of TOR signalling with rapamycin, a TORC1 inhibitor, mimics the impact of nutrient stress to reduce cell size, both in *S*. *pombe* [6] and mammalian cells [9].

The mechanism by which cells sense changes in nitrogen quality is distinct from the means by which changes in amino acid or carbon are sensed [1, 7]. Although a number of studies have focused on identifying and characterising upstream regulators of TORC1 that respond to amino acid or glucose availability [10, 11], little is known about how cells sense nitrogen quality, and how this signal is relayed to TORC1 to adjust cell size accordingly. Recently, we have shown that nitrogen stress-induced TORC1 inhibition requires the Ssp2^AMPKα^ kinase to inhibit TORC1, and that this control also requires Tsc1/2 complex and Rhb1^Rheb^ GTPase [7]; however, the response of *ssp2*^*AMPKα*^.*Δ* (gene deletion) cells to nitrogen stress was significantly reduced, but not completely abolished [7]. Thus, there appear to be multiple layers of TORC1 regulation following nitrogen stress.

PIKFYVE is a 1-phosphatidylinositol-3-phosphate 5-kinase that is required for the production of a signalling phospholipid required for vacuole functions and endosome dynamics, phosphatidylinositol-3,5-bisphosphate (PI(3,5)P_2_) [12]. Recently, PI(3,5)P_2_ was reported to be a positive regulator of TORC1 activity on the yeast vacuole, that was required for TORC1 inhibition of autophagy under nutrient-rich conditions [13]. PIKFYVE also regulates cell type-specific activation and localization of mTORC1 in 3T3-L1 adipocytes [14]. In humans, mutations predicted to lead to minor changes in PI(3,5)P_2_ levels are associated with severe neurological diseases [15] and are implicated in the invasive behaviour of cancer cells [16].

Here we report a novel function for the fission yeast PIKFYVE kinase Ste12^PIKFYVE^, in the regulation of mitotic commitment. A genetic screen identified a non-functional *ste12*^*PIKFYVE*^ mutant that was unable to invoke the normal advancement of mitotic onset and adjust cell size at division in response to nitrogen stress.

## Materials and methods

### Yeast cell cultures & reagents

*S*. *pombe* strains used in this study are listed in S1 Table. Cell growth and maintenance was according to [17]. Liquid cultures were grown exponentially for 48 h at 28°C in YES or in Edinburgh minimal medium 2 (EMM2-N; ForMedium) supplemented with 20mM L-glutamate (EMMG) or 93.5 mM NH_4_^+^ (EMM). For nitrogen downshifts, early exponential cultures of 1.5 x 10^6^ cells/ml in EMMG were filtered into EMMP (EMM2-N + 20mM proline). Cells were either fixed for microscopy, or harvested for biochemistry. For cell growth assays, cells were grown exponentially for 48 hours to 2.5 x 10^6^ cells/ml. A 10-fold dilution series was spotted on indicated plates. Torin1 was added at a concentration of 5 μM (3 μg/ml), rapamycin was added at 300 ng/ml, phloxine B was added at 1g/L. *S*. *pombe* Mip1^RAPTOR^ was tagged endogenously with C-terminal GFP(S65T) as described in [18]

### Genetic screen

Wild-type cultures were grown exponentially for 48 at 28°C in YES. Cells were then plated on EMMP + phloxine B to a density of approximately 2000 cells per plate (200000 cells were screened). Plates were irradiated with UV dose of 0.015J to achieve approx. 60% killing. Dark red colonies were then transferred to EMMG + phloxine, colonies that displayed the same colour as wild-type control were taken forward (the aimed was to identify mutants that were dark only under nutrient poor condition). 50 colonies were selected for secondary screen, cells were grown exponentially in EMMG for 48 hours and then washed twice with EMMP and grown in EMMP for further 120 min. 3.6 x 10^6^ cells were harvested for forward scatter analysis before and at the end of the media shift. Samples were washed in 1 x PBS and cell size was measured using forward scatter analysis (minimum of 30,000 cells per sample). Cell size data were analysed using Summit (Beckman Coulter). 3 candidates that did not reduce cell size at division during the media shift were subsequently backcrossed 3X by tetrad dissection. 1 mutant that showed a 2:2 Mendelian segregation and thus contained a single mutation was subjected to Next Generation Sequencing.

### Protein extraction & western blotting

For Western blotting of *S*. *pombe* cells, total protein extracts were prepared from 6 x 10^7^ cells/sample by precipitation with TCA as previously described [19]. PVDF membrane (Millipore, Co. Durham) and secondary antibodies linked to alkaline phosphatase (AP; Sigma-Aldrich, Dorset) were used. Primary antibodies were: anti-TAT1 (1:2000; kind gift from Keith Gull); anti-Tor1, Anti-Tor2, anti-phospho-Tor2 S1975 and anti-phospho Tor1 T1972 (1:100 [20]); anti-Gad8 (1:150), anti-phosho-Gad8-S546 (1:1000) and anti-phospho-Gad8-T387 (1:100, [20]); anti-PK-Tag (1:2000; AbD Serotec, Oxford); anti-eIF2α (1:500, Santa Cruz Biotechnology); anti-phospho-eIF2α S51 (1:2000; Millipore). To calculate average signal intensities on western blots, student’s t-tests were carried out using GraphPad Prism 6.0 software.

### Tor1 and Tor2 immunoprecipitation

For Tor1 or Tor2 IP, 3x10^8^ glutamine grown cells (culture density 2,4x10^6^ cells ml^-1^) were harvested and re-suspended in IP buffer (50 mM HEPES pH 7.5, 150 mM NaCl, 0,1% CHAPS, 0,05% Tween20, 50 mM L-arginine, 50 mM L-glutamic acid, 50 mM NaF, 2 mM NaVO_4_, 60 mM sodium glycerol phosphate, 5 mM NEM, 1 mM PMSF, 1 mM DTT and Sigma protease inhibitor cocktail) and broken in a FastPrep using glass beads. The cell extract was incubated with Invitrogen protein Protein G Dynabeads pre-coupled with anti-Tor1, or anti-Tor1 antibodies [20] for 20 min at 4°C. Beads were then washed three times with IP buffer and heated to 80°C for 10 min to elute the proteins. Samples were loaded on a SDS-PAGE gel and subsequently processed as total protein extracts.

### Microscopy

For septation/mitotic index, cells were fixed with 37% formaldehyde (final 10% v/v) and washed with 1 x PBS. Septa were stained with calcofluor white (Sigma-Aldrich). For mitotic index, cells were after formaldehyde fixation washed in cold 70% ethanol and incubated for a minimum of 5 minutes on ice, then washed 2x with PBS and stained with calcofluor white and DAPI (Sigma-Aldrich) to stain both septa and nuclei. 300 cells were counted per time point and 100 dividing cells were measured for cell length using ImageJ. To calculate significant differences between average cell lengths student’s t-tests were carried out using GraphPad Prism 6.0 software. To image vacuoles, cells were incubated for 30 min with FM4-64 in DMSO (8 μM; VWR International). The diameters of vacuoles were measured using ImageJ. Live cell imaging was conducted with a Spinning disk confocal fluorescent microscope with CMOS camera (PerkinElmer).

### Autophagy assay

A failure to survive nitrogen starvation can be used as a proxy for autophagy defects. Cells were grown exponentially at 28°C in EMM for 48 hours. *leu1*.*32* cultures were supplemented with 0.015 mg/ml Leucine. Cells were then washed twice in EMM2-N, re-suspended in EMM2-N and grown for the indicated times. A 10-fold dilution series was spotted on YES plates to calculate survival.

### SILAC cell culture

*car2*::*NAT lys1-131 arg3-d4* cells were inoculated in YES media overnight and then washed into EMM-G containing 75mg/L of either light (l-arginine monohydrochloride (Sigma) and l-lysine monohydrochloride (Sigma)) or medium ((LYSINE-L, 2HCl 4.4.5.5-D4 (Cat code DLM-2640, Euroisotop), ARGININE-L, HCl, U-13C6 99%13C (Cat code CLM-2265, Eurisotop,)) amino acids. Cells were cultured in log phase for 48 hours to ensure complete incorporation of labelled amino acids into the proteome. Light labelled cultures were treated with DMSO and medium labelled cultures were treated with a final concentration of 25 μM Torin1 at a density of 2.04 x 10^6^ cells/ml. Approximately 4.8 x 10^9^ cells were harvested for each sample. After 30 minutes cultures were harvested by centrifugation, washed in 20ml of STOP buffer (10 mM EDTA, 1 mM sodium azide, 50 mM sodium fluoride (NaF), 0.9% NaCl), followed by washing with 10ml of ice cold ddH_2_0. The final pellets were then resuspended in an appropriate volume of ice cold ddH_2_0 and dropped directly into liquid nitrogen to produce frozen cell droplets.

### SILAC protein extraction

Samples were processed using a SPEX Sample Prep LLC 6850 Freezer Mill in presence of liquid nitrogen. The resulting cell powder was resuspended in denaturation buffer (6M urea, 2M thiourea, 1% n-octyl glucoside) at a ratio of 500mg powder to 500μl denaturation buffer. Insoluble material was removed by centrifugation (13,000 g, 10 minutes at 4°C) and the supernatant was designated supernatant I (soluble fraction). The pellet was then resuspended in 500μl denaturation buffer, 500μl glass beads were added and then subjected to 20 seconds shaking in a FastPrep machine (FP120, Qbiogene). The resulting suspension was again centrifuged (13,000 g, 10 minutes at 4°C) and the supernatant retained (supernatant II). The pellet was then discarded. Protein concentrations were determined by Bradford assay according to manufacturers instructions. The supernantants were process for Mass spectrometry as described previously in Barker et al 2016 [21].

## Results

### Ste12 is required for nitrogen stress-induced cell size control

**Fig 1:**
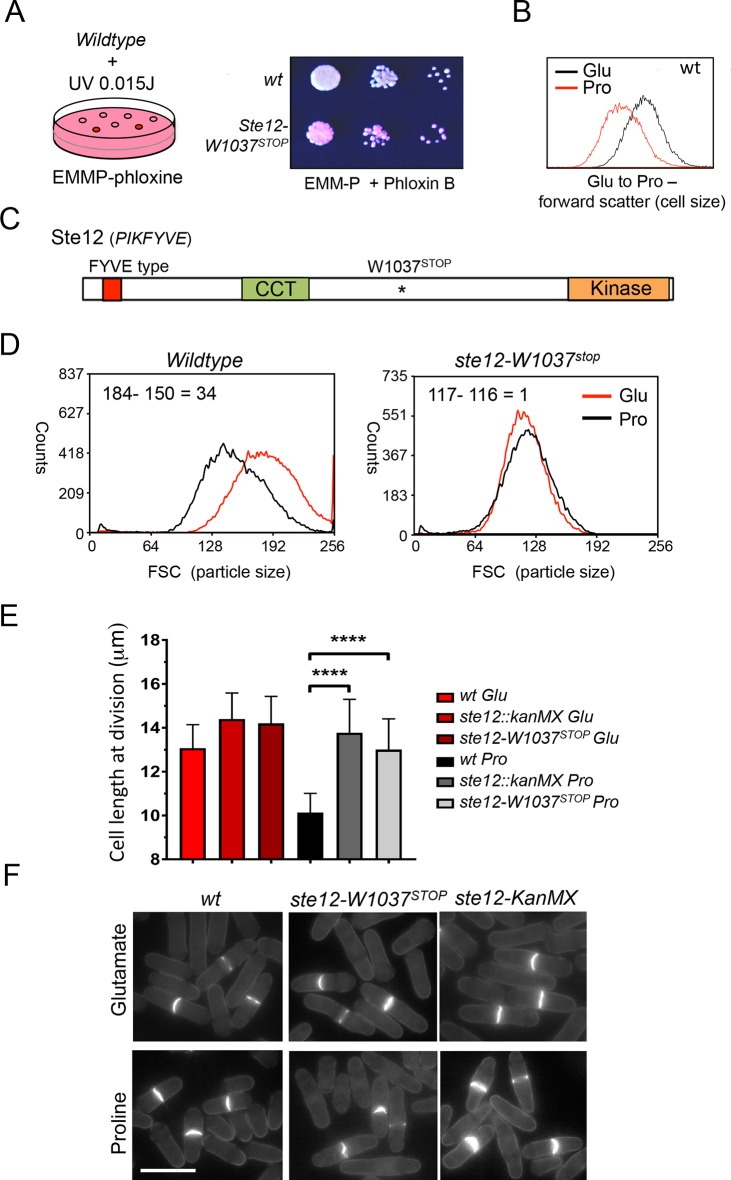
A genetic screen for nitrogen-sensing mutants identified *ste12*^*PIKFYVE*^*-W1037*^*STOP*^. (A) Early exponential EMMG wild-type cultures were plated on EMMP + phloxine plates and irradiated to induce random mutagenesis. Dark red colonies that maintained the same colour as wild-type cells on EMMG + phloxin and EMMG + phloxin + rapamycin were then taken forward for a EMMG to EMMP media shift for forward scatter analysis to measure cell size. Glu = glutamate; pro = proline. (B) *ste12*^*PIKFYVE*^*-W1037*^*STOP*^ cells appear darker red on EMMP + phloxin than wild-type cells. (C) Schematic representation of *ste12*^*PIKFYVE*^*-W1037*^*STOP*^. FYVE, Fab1-YOTB-Vac1-EEA1-type domain; CCT, Chaperonin-Containing-T-complex domain. (D-F) *ste12*^*PIKFYVE*^*-W1037*^*STOP*^ cells are unable to reduce cell size when shifted from EMMG to EMMP as measured with forward scatter (D) (note the use of light scattering is not ideal if strains of different genotypes are compared. This can be seen here, e.g. the large vacuoles of *ste12* (see Fig 2) altered the light scattering and therefore the relative “size”, despite the fact that *ste12* mutant and wild type cells are the same cell size), cell length measurements (E) and images of dividing cells (F).

Nitrogen stress alters the timing of mitotic commitment in fission yeast, such that a reduction in nitrogen quality accelerates mitotic commitment, leading to a concomitant reduction in cell size at division (Fantes and Nurse, 1977). We performed a genetic screen to identify mutants defective in nitrogen sensing. Lawns of prototrophic single cells were exposed to random mutagenesis by exposure to ultraviolet light. Surviving single cells were grown to small size colonies in the presence of a poor nitrogen source (proline) as their only source of nitrogen. Under such conditions, wild type cells propagate at a size that is shorter than when grown in the presence of a good nitrogen source (glutamate) [1, 7, 22]. Addition of phloxine B (red dye) to the proline based media identified dark pink mutant colonies, in which an elevated number of dying (permeable) or larger cells took up more dye than neighbouring healthy colonies. Visual screening of 40 candidates identified 1 viable mutant that was unable to reduce cell size at division upon exposed to nitrogen stress in liquid media (Fig 1A and 1B). Next Generation Sequencing revealed a truncating, premature stop codon at position W1037 of the Ste12^PIKFYVE^ -phosphatidylinositol-3-phosphate 5-kinase (Fig 1C) [12, 23]. Forward scatter FACS analysis confirmed that *ste12*^*PIKFYVE*^.*W1037*^*STOP*^ cells failed to reduce cell size at division when shifted from a good nitrogen source (glutamate) to a poor nitrogen source (proline) (Fig 1D, 1E and 1F).

**Fig 2:**
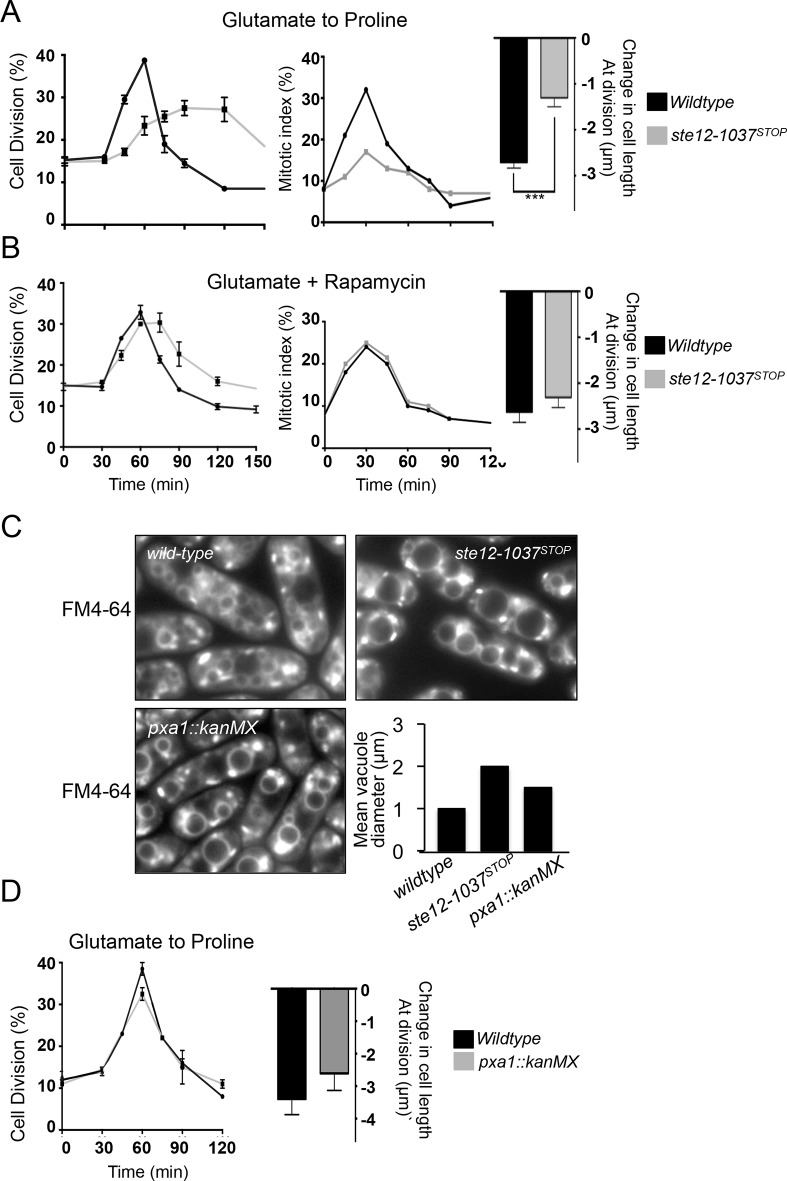
Ste12 may act upstream of TORC1. Altered vacuole size defects do not account for the nitrogen stress-response defect of *ste12*^*PIKFYVE*^.*W1037*^*STOP*^ mutant. (A,B) Nitrogen stress (upper panel) and rapamycin (bottom panel) response of *ste12*^*PIKFYVE*^*-W1037*^*STOP*^ and wild-type cells. The frequencies of mitotic and dividing cells along with changes in cell length at division are shown (C) Cells grown exponentially at 28°C in EMMG were stained with FM4-64. *ste12*^*PIKFYVE*^.*W1037*^*STOP*^ and an alternative vacuole mutant *pxa1*.*Δ* exhibit enlarged vacuoles compared to wild-type. (D) *pxa1*.*Δ* can respond to nitrogen stress as efficiently as wild type.

In wild-type cultures, nitrogen stress promotes mitosis and cell division to invoke a transient sharp increase in the frequency of dividing cells that is accompanied by a reduction in cell length at division (cell size) (Fig 2A). In contrast, the advancement of cell division was markedly compromised in *ste12*^*PIKFYVE*^.*W1037*^*STOP*^ cells as a consequence of a reduction in the efficiency with which mitosis was advanced by the stress (Fig 2B). Thus, *ste12*^*PIKFYVE*^.*W1037*^*STOP*^ cells were deficient in their nitrogen stress response.

### Ste12 may act upstream of TORC1

Nitrogen stress inhibits TORC1 signalling to accelerate mitotic commitment [6, 7]. Chemical inhibition of TORC1 by rapamycin in steady state good quality nitrogen cultures has the same impact on cell size control as nitrogen stress [6]. We inhibited TORC1 directly by the addition of rapamycin to *ste12*^*PIKFYVE*^.*W1037*^*STOP*^ cells to determine the relative position of Ste12 in the nitrogen stress signalling cascade. Rapamycin rescued both the inability to advance mitosis and reduce cell size at division (Fig 2B), suggesting that Ste12 may acts upstream of TORC1.

### Altered vacuole size of *ste12*^*PIKFYVE*^*-W1037*^*STOP*^ is unlikely to be the determinant of nitrogen-dependent cell size defects

Ste12 and its product, PI(3,5)P_2_, is important for the maintenance of vacuole size [12, 23]. To examine the sizes of vacuoles in *ste12*^*PIKFYVE*^.*W1037*^*STOP*^, we used FM4-64, a lipophilic vital dye that binds non-specifically to the endocytic pathway in living cells [24]. In steady state growth conditions, *ste12*.*W1037*^*STOP*^ vacuoles labelled with FM4-64 were approximately twice as large as the vacuoles of wild-type cells (Fig 2C).

To explore the possibility that the enlarged vacuoles seen in *ste12*^*PIKFYVE*^.*W1037*^*STOP*^ mutants prevent the control of mitosis and cell size upon nitrogen stress, we examined the response of an alternative vacuole size mutant, *pxa1*.*Δ*. Pxa1 is required for normal vacuole function and morphology in *S*. *pombe*, and *pxa1*.*Δ* cells possess large vacuoles [25]. However, Pxa1 function is different to that of Ste12, as Pxa1 is not involved in the production of PI(3,5)P_2_. Despite having enlarged vacuoles (Fig 2C), *pxa1*.*Δ* cells were able to respond to nitrogen stress to the same degree as wild-type cells (Fig 2D). We conclude that altered vacuole size is unlikely to be responsible for the inability of *ste12*^*PIKFYVE*^.*W1037*^*STOP*^ cells to advance mitosis in response to nitrogen stress.

### *ste12*^*PIKFYVE*^*-W1037*^*STOP*^ autophagy defect is unlikely to account for the inability to control size after nitrogen stress

**Fig 3:**
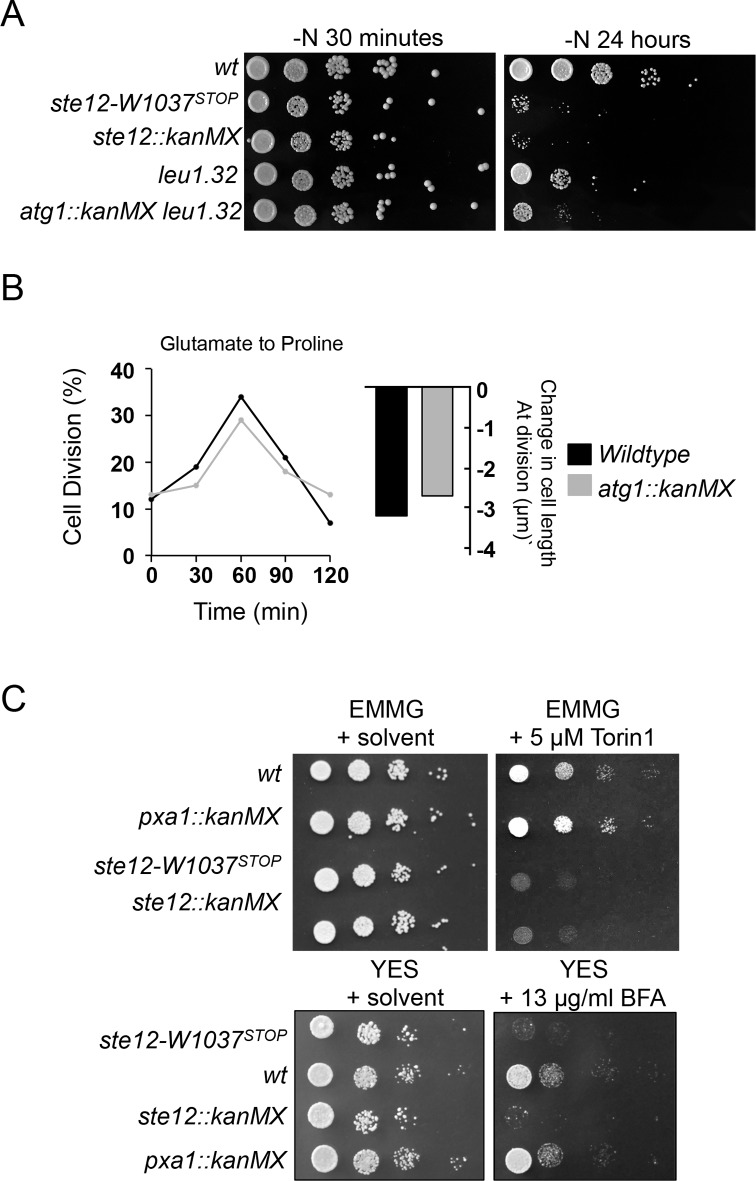
Autophagy defects is unlikely to account for the nitrogen stress-response defect of *ste12*^*PIKFYVE*^.*W1037*^*STOP*^ mutant. (A) *ste12*^*PIKFYVE*^.*W1037*^*STOP*^ cells are autophagy-deficient. Cells were starved of nitrogen for the indicated times and then plated on YES media as 10-fold serial dilutions. (B) Autophagy-deficient *atg1*.*Δ* can respond to nitrogen stress as efficiently as wild type. (C) Ste12-defficient strains are sensitive to Torin1 and Brefeldin A. Exponentially growing cultures in EMMG were plated on indicated media as 10-fold serial dilutions.

Previous studies have shown that the *Drosophila* Fab1^PIKFYVE^ is required for autophagy, more specifically, for proper maturation of the autolysosomes that are formed after the fusion of lysosomes to autophagosomes [26]. A similar autophagy deficiency has been observed when budding yeast can not make PI(3,5)P_2_ [13]. Autophagy is linked to the response to nitrogen starvation, as cells induce autophagy to allow survival under periods where energy levels are low [27]. Autophagy-related 1 (Atg1) kinase is essential for autophagosome formation, and *S*. *pombe atg1*.*Δ* cells are unable to induce autophagy [28]. A failure to survive nitrogen starvation can be used as a proxy for autophagy defects. Both *ste12*^*PIKFYVE*^.*W1037*^*STOP*^ and *ste12*^*PIKFYVE*^.*Δ* cells may be compromised in their ability to induce autophagy because they resemble *atg1*.*Δ* in being defective in recovery after prolonged nitrogen starvation (Fig 3A).

We next asked whether defective autophagy accounts for the inability of *ste12*^*PIKFYVE*^.*W1037*^*STOP*^ to advance mitosis and reduce cell size after nitrogen stress. The autophagy mutant *atg1*.*Δ* advanced mitotic commitment and reduced cell size after nitrogen shift as proficiently as wild type cells (Fig 3B), indicating that autophagy is not required for the nitrogen stress response.

### No significant changes in TORC1 and TORC2 activities were observed in *ste12*^*PIKFYVE*^*-W1037*^*STOP*^ mutants

**Fig 4:**
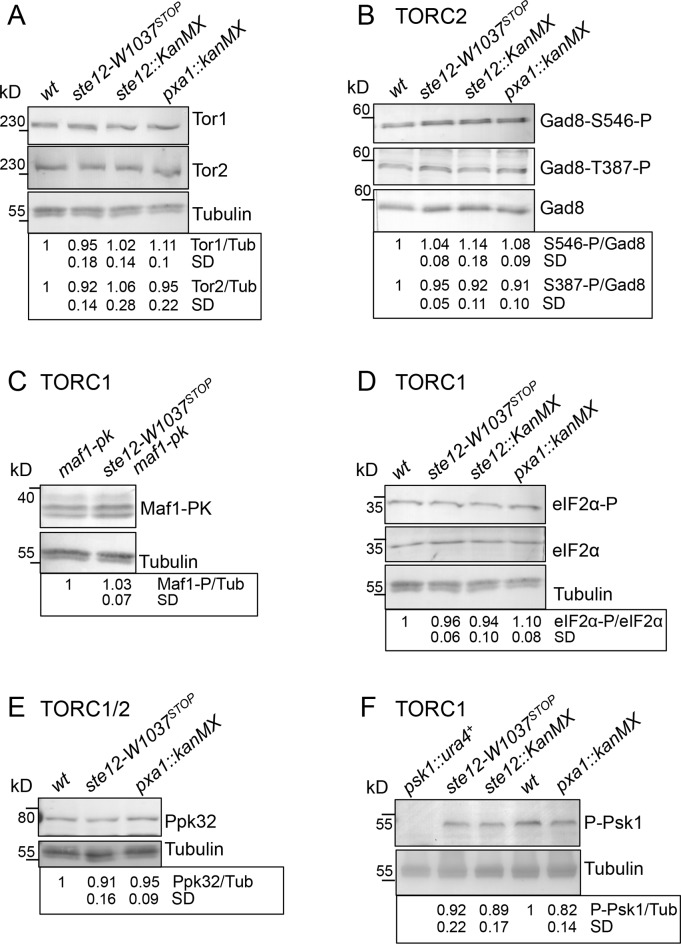
TORC1 and TORC2 activities are not significantly changed in *ste12*^*PIKFYVE*^*-W1037*^*STOP*^. (A-F) Assessment of TORC1/2 *in vivo* activity in steady-state EMMG cultures by immunoblotting of known previously established substrates. All antibodies and Maf1.PK have been validated previously in Du, et al 2012 and Halova et al 2013.

Recent work has suggested that PI(3,5)P_2_ regulates TORC1 activity [13, 14]. Furthermore, addition of rapamycin to *ste12*^*PIKFYVE*^.*W1037*^*STOP*^ cells rescued their inability to advance mitosis and reduce cell size at division (Fig 2B). These phenotypes suggest that *ste12*^*PIKFYVE*^.*W1037*^*STOP*^ cells have deregulated TORC1 signalling. Interestingly, in steady state growth conditions, both *ste12*^*PIKFYVE*^.*W1037*^*STOP*^ and *ste12*^*PIKFYVE*^.*Δ* mutants, but not *pxa1*.*Δ* (included to control for the impact that enlarged vacuoles can have on TOR activity), exhibited a sensitivity to low concentrations of the ATP-competitive TOR inhibitor, Torin1 (Fig 3C) that suggests that they may have compromised TOR activity. Alternatively, sensitivity to Torin1 could arise from altered uptake of the drug in the *ste12*^*PIKFYVE*^ mutant. The response to rapamycin of the *ste12*^*PIKFYVE*^.*W1037*^*STOP*^ mutant was identical to that of wild type (Fig 2B), suggesting that the Torin1 sensitivity may be a specific response. However, *ste12*^*PIKFYVE*^.*Δ* mutants were also sensitivity to Brefeldin A (induces ER and Golgi stress) (Fig 3C), therefore, *ste12* mutants may be hypersensitive to several drugs. Alteration of the levels of the TOR kinases do not account for the Torin1 sensitivity because Tor1 and Tor2 protein levels were unchanged by the inclusion of the *ste12*.*W1037*^*STOP*^ and *ste12*^*PIKFYVE*^.*Δ* allele (Fig 4A). Maf1 is a repressor of RNA polymerase III, that is a TORC1-specific substrate whose migration in SDS PAGE is retarded by phosphorylation. A reduction in TORC1 activity towards Maf1 leads to a collapse in the slower migrating phospho-forms of Maf1 [29]. Phosphorylation of the eukaryotic initiation factor 2α (eIF2α) is an alternative read-out of TORC1 activity. In *S*. *pombe*, inhibition of TORC1 promotes eIF2α phosphorylation on Ser52 in a Gcn2-dependent manner [29–31]. No change in either Maf1 or eIF2α phosphorylation status was observed in cells lacking functional Ste12^PIKFYVE^ (Fig 4C and 4D). The phosphorylation status of the AGC kinase Psk1 and level of Ppk32 are further readouts of TORC1 signalling [32, 33]. Mutation of *ste12* had no significant impact upon either readout of TORC1 activity (Fig 4E and 4F). Although not significant, the Psk1 phosphorylation did appear slightly reduced in both *ste12* and *pxa1* mutants. However the *pxa1* mutant was not sensitive to TOR inhibition (Fig 3C). Furthermore, *pxa1 and psk1* mutants were both able to respond to nitrogen stress to the same degree as wild-type cells (Fig 2D & S2 Fig). The phosphorylation status of the TORC2 substrate Gad8 on S546 and the Ksg1^PDK1^ dependent activating phosphorylation of Gad8-T387 [34, 35], also remained unchanged (Fig 4B).

Together, these observations indicate that normal TORC1 and TORC2 activities are maintained in the *ste12* mutants. The autophagy deficient *atg20* mutant is sensitive to altered TOR activity [36]. Therefore, the observed sensitivity to Torin1 (Fig 3C) of *ste12* mutants may arise from their participation in autophagy or it may represent a general hypersensitivity to drugs, rather than a symptom of the changes in cell cycle control.

### TORC1 localizes to vacuoles in both wild type and *ste12*^*PIKFYVE*^.*W1037*^*STOP*^

**Fig 5:**
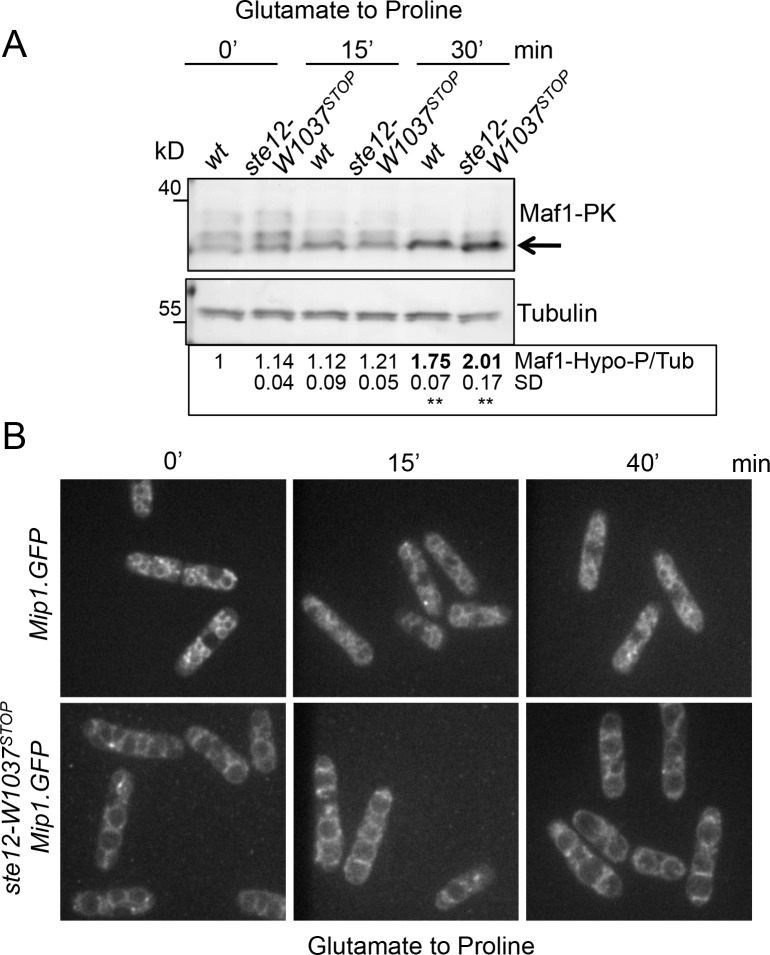
TORC1 remains on vacuoles during nitrogen stress in both wild type and *ste12*^*PIKFYVE*^.*W1037*^*STOP*^. (A) Western blot of protein extracts from exponentially grown cells. TORC1 activity of *ste12*^*PIKFYVE*^.*W1037*^*STOP*^ is inhibited by nitrogen stress similarly as in control cells as determined by immunoblotting for Maf1 phosphorylation. (B) Live cell imaging at indicated time-points of exponentially growing cultures shifted from media containing glutamate to proline as a nitrogen source. Mip1^RAPTOR^.GFP remains localized to vacuoles in the *ste12*^*PIKFYVE*^.*W1037*^*STOP*^ mutant.

As mentioned above, nitrogen stress inhibits TORC1 (but not TORC2) activity to advance mitotic onset and thereby reduce cell size at division [6, 7]. We therefore determined whether *ste12*^*PIKFYVE*^.*W1037*^*STOP*^ cells were capable of down-regulating TORC1 activity under nitrogen stress by monitoring Maf1 phosphorylation during a glutamate to proline nitrogen shift. Both the wild-type control strain and the *ste12*^*PIKFYVE*^.*W1037*^*STOP*^ mutant down-regulated Maf1 phosphorylation within 15 minutes of the nitrogen shift (Fig 5A) to indicate that TORC1 activity towards Maf1 is unaltered in *ste12*^*PIKFYVE*^.*W1037*^*STOP*^ cells.

We next set out to monitor TORC1 localization in the *ste12*^*PIKFYVE*^.*W1037*^*STOP*^ mutant. The vacuole/lysosome is a site of mTORC1 activation in mammalian cells [10]. TORC1 localization to the vacuoles has also been observed in fission yeast [37]. Interestingly, PIKFYVE regulation of mTORC1 localization was recently reported in 3T3-L1 adipose cells [14]. The localization of Mip1^RAPTOR^ in *ste12*^*PIKFYVE*^*-W1037*^*STOP*^ *mip1*^*RAPTOR*^.*GFP* cells revealed no obvious changes in the recruitment of TORC1 to vacuoles throughout the nitrogen shift (Fig 5B). To ensure that tagging Mip1^RAPTOR^ with GFP did not alter protein function we monitored the phosphorylation of TORC1 substrates and cell size control during nitrogen stress (S1 Fig). Neither function was altered in the Mip1^RAPTOR^.GFP cells. In summary, we did not observe any major alterations in TORC1 localization in *ste12*^*PIKFYVE*^.*W1037*^*STOP*^ mutant, although we cannot rule out differences that are below the resolution of our imaging systems, which may have a significant impact on localized TORC1 activity.

### Gad8 contributes to nitrogen-regulated commitment of mitosis.

**Fig 6:**
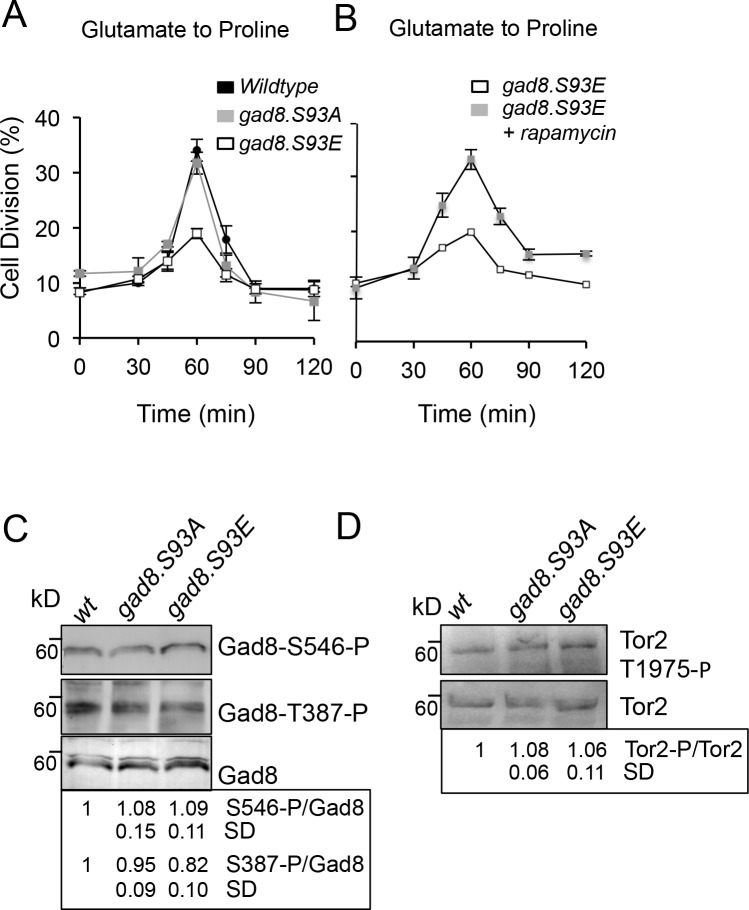
Gad8 contributes to nitrogen-regulated commitment of mitosis. (A,B) Western blot of protein extracts from exponentially grown cells. (C,D) Exponentially growing cultures shifted from media containing glutamate to proline as a nitrogen source and the numbers of dividing cells were counted at indicated time points.

We previously reported the requirement for the *S*. *pombe* AGC kinase, Gad8, for the nitrogen stress response [34, 38]. This role is specific to the Gad8 AGC kinase, as none of the 3 other known AGC kinases in fission yeast are required (S2 Fig). To gain additional insight into nitrogen dependent regulation of Gad8, we examined data from a SILAC (Stable isotope labelling by amino acids in cell culture) mass spectrometric screen for phospho-peptides that showed differential phosphorylation upon nitrogen stress or Torin1 inhibition of TOR function [39]. A novel phosphorylation site on Gad8 Serine 93 was down-regulated more than 4 fold in response to nitrogen stress and TOR inhibition (S3 Fig). Interestingly, this phosphorylation site may be conserved in human AKT2 and AKT3 (S3 Fig). To establish whether Gad8.S93 phosphorylation alters the response to nitrogen stress we generated strains in which the endogenous Gad8 locus was mutated to encode a Gad8 protein in which serine 93 had been replaced with alanine to block phosphorylation or to glutamic acid in an attempt to mimic phosphorylated serine. Ksg1^PDK1^ and TORC2 dependent activation of Gad8 was not significantly affected by the Gad8 serine 93 mutants (Fig 6A). However, *gad8*.*S93E* mutant cells (unable to down-regulate S93 phosphorylation) were unable to advance into mitosis as efficiently as wild-type and *gad8*.*S93A* cells (Fig 6C). Importantly, rapamycin treated *gad8*.*S93E* mutants advanced into mitosis with the same kinetics as wild-type cells (Fig 6D) to suggest that the requirement for a reduction in Gad8.S93 phosphorylation sits upstream of TORC1 in the nitrogen stress response.

### Gad8 mediated TORC1 phosphorylation of Tor2.S1975 upon nitrogen stress is insensitive to changes in Ste12^*PIKFYVE*^ function

**Fig 7:**
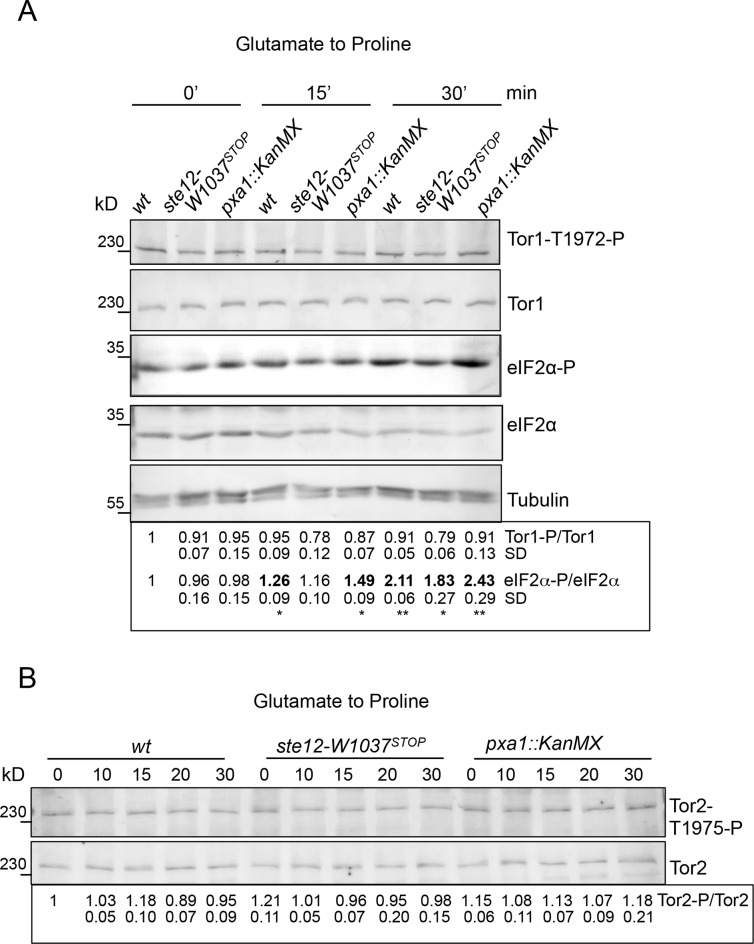
Gad8 mediated TORC1 phosphorylation of Tor2.S1975 is not altered upon nitrogen stress. (A-B) Western blots of protein extracts from nitrogen stressed cells show that Gad8-mediated Tor1.T1972 or Tor2.S1975 phosphorylation is not affected in the *ste12* mutant.

Gad8 phosphorylates Tor1 (as part of TORC2) on Thr1972 to inhibit TORC2 activity when cells are exposed nitrogen starvation [20]. Importantly, Gad8 also phosphorylates the analogous residue of Tor2 (Tor2-S1975) [20]. We therefore monitored the impact of *ste12*^*PIKFYVE*^.*W1037*^*STOP*^ upon the dynamics of Tor1-T1972 and Tor2-S1975 phosphorylation during nitrogen stress to determine whether Gad8 dependent control of TOR activities was altered in the *ste12* mutant. In both mutant and wild-type cells, Tor1-T1972 and Tor2-S1975 phosphorylation remained constant throughout the course of the nitrogen down-shift (Fig 7A and 7B). Tor1-T1972 and Tor2-S1975 phosphorylation also remained unaffected by the *gad8*.*S93E* and *gad8*.*S93A* mutations (Fig 6B). Importantly, the increased eIF2α phosphorylation known to be associated with reduced TORC1 activity was observed following the nitrogen stress (Fig 7A). Together, these data suggest that Gad8 dependent Tor2-S1975 phosphorylation is unlikely to account for TORC1 inhibition upon nitrogen stress in wild type cells.

## Discussion

Environmental control of cell division, and therefore cell size, has been observed in all eukaryotes studied. Limitations in nutritional environment restrain protein synthesis to conserve crucial metabolites and consequently, promote cell division to reduce size. The reduction in cell size reduces the demand for protein synthesis in each consecutive cell cycle. We performed a genetic screen for nitrogen-sensing mutants, to gain further, unbiased, insight into the regulation of cell division and cell size in response to the changes in the environment. We identified a mutation in the fission yeast 1-phosphatidylinositol-3-phosphate 5-kinase Ste12 (*ste12*^*PIKFYVE*^.*W1037*^*STOP*^). Ste12^PIKFYVE^ and its product, PI(3,5)P_2_, are required for vacuole functions and endosome dynamics in protein trafficking [12, 23]. We now describe a role of Ste12^PIKFYVE^, in regulating cell division and cell size in response to changes in the quality of available nitrogen. Although *ste12*^*PIKFYVE*^.*W1037*^*STOP*^ mutant cells maintain a wild-type cell size when grown in a good nitrogen source (glutamate) (Fig 1E and 1F), they were unable to reduce their cell size when the quality of the nitrogen source changed to poor (proline).

As for cells lacking the equivalent kinase in budding yeast [13], *ste12*^*PIKFYVE*^.*W1037*^*STOP*^ cells displayed enlarged vacuoles (Fig 2C) and potential autophagy defect (Fig 3A). The altered vacuole morphology however is unlikely to account for the compromised response to nitrogen stress, as an alternative vacuole size mutant, *pxa1*.*Δ*, was fully proficient in reducing cell size at division (Fig 3D). Furthermore, the autophagy defect of *ste12*^*PIKFYVE*^*-W1037*^*STOP*^ is also unlikely to be responsible for its inability to reduce cell size at division under nitrogen stress either, as the response of an autophagy-deficient *atg1*.*Δ* strain to nitrogen stress was indistinguishable from that of wild type cells (Fig 3B).

We previously established that TOR inhibition was required for nitrogen-stress induced advance of mitotic onset to reduce cell size at division [6]. The addition of rapamycin to Ste12^PIKFYVE^ deficient mutants, to inhibit TORC1 directly, (Fig 2B) established that Ste12^PIKFYVE^ deficient mutants can respond to TORC1 inhibition and advance mitosis to reduce cell size. This suggests that Ste12^PIKFYVE^ either acts upstream of TORC1 and fails to fully inhibit TORC1 activity following nitrogen stress or alternatively, Ste12 regulates cell size at division in a TORC1 independent and unknown manner.

PI(3,5)P_2_ lipids have recently been associated with TORC1 signalling in both budding yeast [13] and adipocytes [14]. In budding yeast, the TORC1 substrate Sch9 AGC kinase, was recruited to the vacuole by PI(3,5)P_2_ were it is activated by TORC1 [13] while mammalian AKT acts on lysosomes [40].

In the Ste12^PIKFYVE^ deficient mutants TORC1 activity towards Maf1, Eif2a, Psk1 and Ppk32 appears to be uncompromised. Furthermore, TORC1 activity towards Maf1 was still inhibited by nitrogen stress in the *ste12*^*PIKFYVE*^.*W1037*^*STOP*^ mutant to the same degree as in wild type cells (Fig 4A). Importantly, despite this nitrogen-stress induced TORC1 inhibition, *ste12*^*PIKFYVE*^.*W1037*^*STOP*^ mutants fail to advance mitotic commitment (Fig 1). As mentioned previously, It is well established that TORC1 acts on vacuoles/lysosomes [41]; it is therefore possible that following nitrogen stress, TORC1 is inhibited locally on the vacuoles, and that it is this localized TORC1 inhibition that is misregulated in the *ste12*^*PIKFYVE*^.*W1037*^*STOP*^ mutant. Gad8 is unlikely to be responsible for such local TORC1 inhibition, because the fission yeast AGC kinase Gad8 localizes to the plasma membrane [42] and in the *ste12*^*PIKFYVE*^.*W1037*^*STOP*^ mutant, Gad8-dependent phosphorylation of Tor2.S1975 was unaffected. Furthermore, in contrast to severe nitrogen starvation, no significant change in inhibitory Gad8-dependent phosphorylation of TORC2 was observed upon nitrogen stress (Fig 6).

In summary, nitrogen-stress controlled TORC1 inhibition advance mitotic onset to reduce cell size [6]. Vacuoles are the site of TORC1 localization and activation [41]. It was previously shown that mutants in PIKFYVE 1-phosphatidylinositol-3-phosphate 5-kinase fission yeast homolog Ste12 have altered and enlarged vacuoles [12, 23]. Here, a genetic screen identified a non-functional *ste12*^*PIKFYVE*^ mutant, which was unable to advance mitotic onset and adjust cell size in response to nitrogen stress. The addition of rapamycin to inhibit TORC1 in Ste12^PIKFYVE^ deficient mutants reduced cell size at division to suggest that Ste12^PIKFYVE^ functions is not required for TORC1 control of mitotic onset to promote cell division and reduce size.

## Supporting information

S1 Fig Mip1^RAPTOR^.GFP is functional. (A) Mip1 protein levels and the ability of TORC1 to phosphorylate downstream targets are not affected in *mip1*^*RAPTOR*^.*GFP*. (B) *mip1*^*RAPTOR*^.*GFP* can respond to nitrogen stress as efficiently as wild type.
(TIF)

S2 Fig The fission AGC kinases Psk1, Sck1 and Sck2 are not required for nitrogen stress induced advancement of mitosis. Exponentially growing cultures were shifted from media containing glutamate to proline as a nitrogen source and the numbers of dividing cells were counted at indicated time points.
(TIF)

S3 Fig Novel phosphorylation of Gad8 serine 93 is reduced upon nitrogen stress or TOR inhibition with Torin1. (A) The spectrum shows the fragmentation pattern of the Gad8 phosphopeptide LPSGHVPANYGVS(ph)IDNSLLAPPLSNGSGHAR indicating S93 to be phosphorylated. The mass of the parent ion is 3176.54043, the measured mass error is 0.12997 ppm. (B) The Gad8.S93 phosphorylation site may be conserved in human AKT2 and AKT3. (C,D) Gad8.S93 phosphorylation is down regulated more that 4 fold upon nitrogen stress or the addition of Torin1. D, SILAC MS spectrum showing that nitrogen stress reduces Gad8.S93 phosphorylation.
(TIF)

S1 Table *S*. *pombe* strains used in this study. (DOCX)
