# Supplementary figures and images for: Ste12/Fab1 phosphatidylinositol-3-phosphate 5-kinase is required for nitrogen-regulated mitotic commitment and cell size control
Source: PLoS One. 2017 Mar 8;12(3):e0172740. doi: 10.1371/journal.pone.0172740 (PMC5342193; doi:10.1371/journal.pone.0172740)

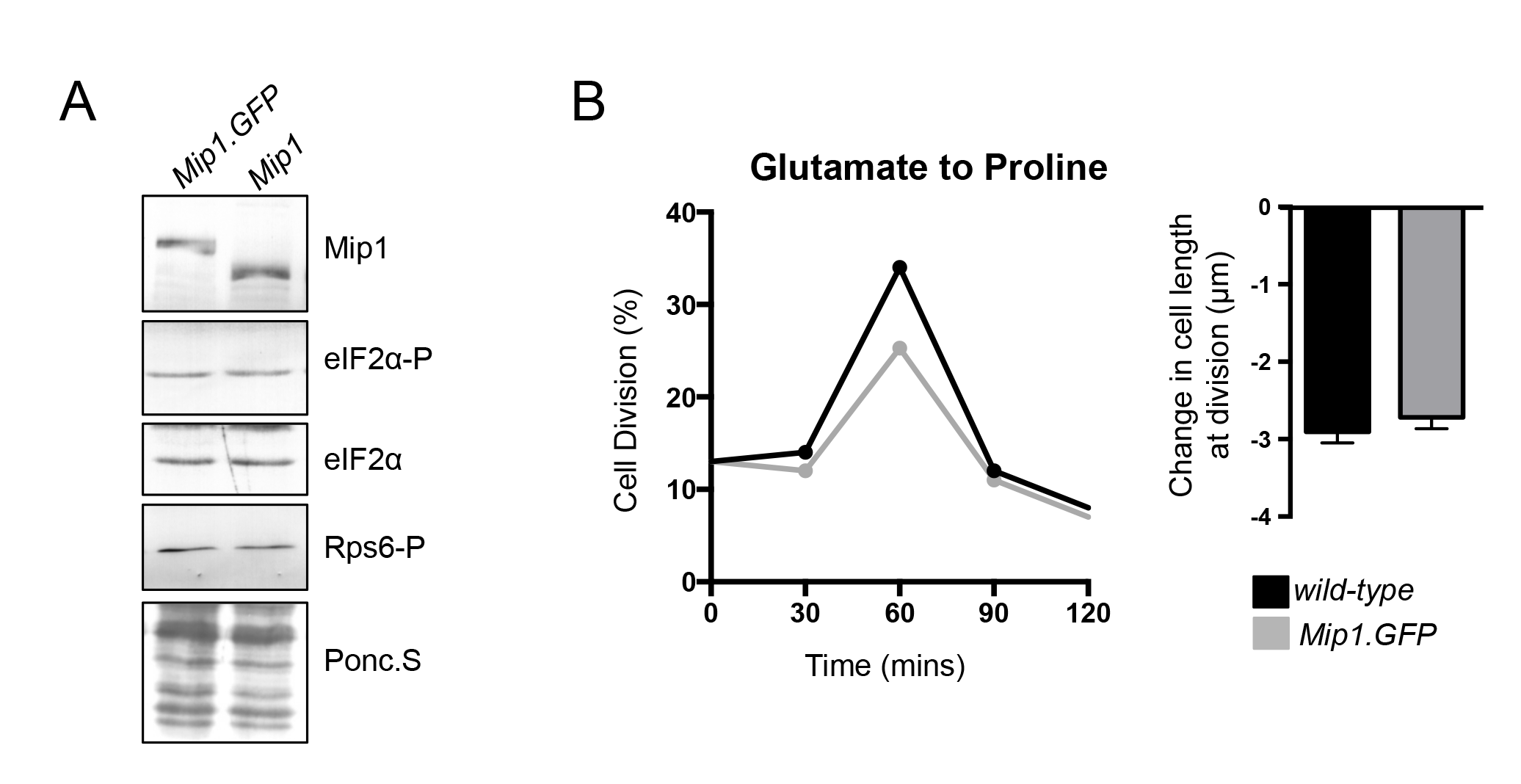

Supplement: S1 Fig — (A) Mip1 protein levels and the ability of TORC1 to phosphorylate downstream targets are not affected in mip1RAPTOR.GFP. (B) mip1RAPTOR.GFP can respond to nitrogen stress as efficiently as wild type. (TIF) [file pone.0172740.s001.tif]

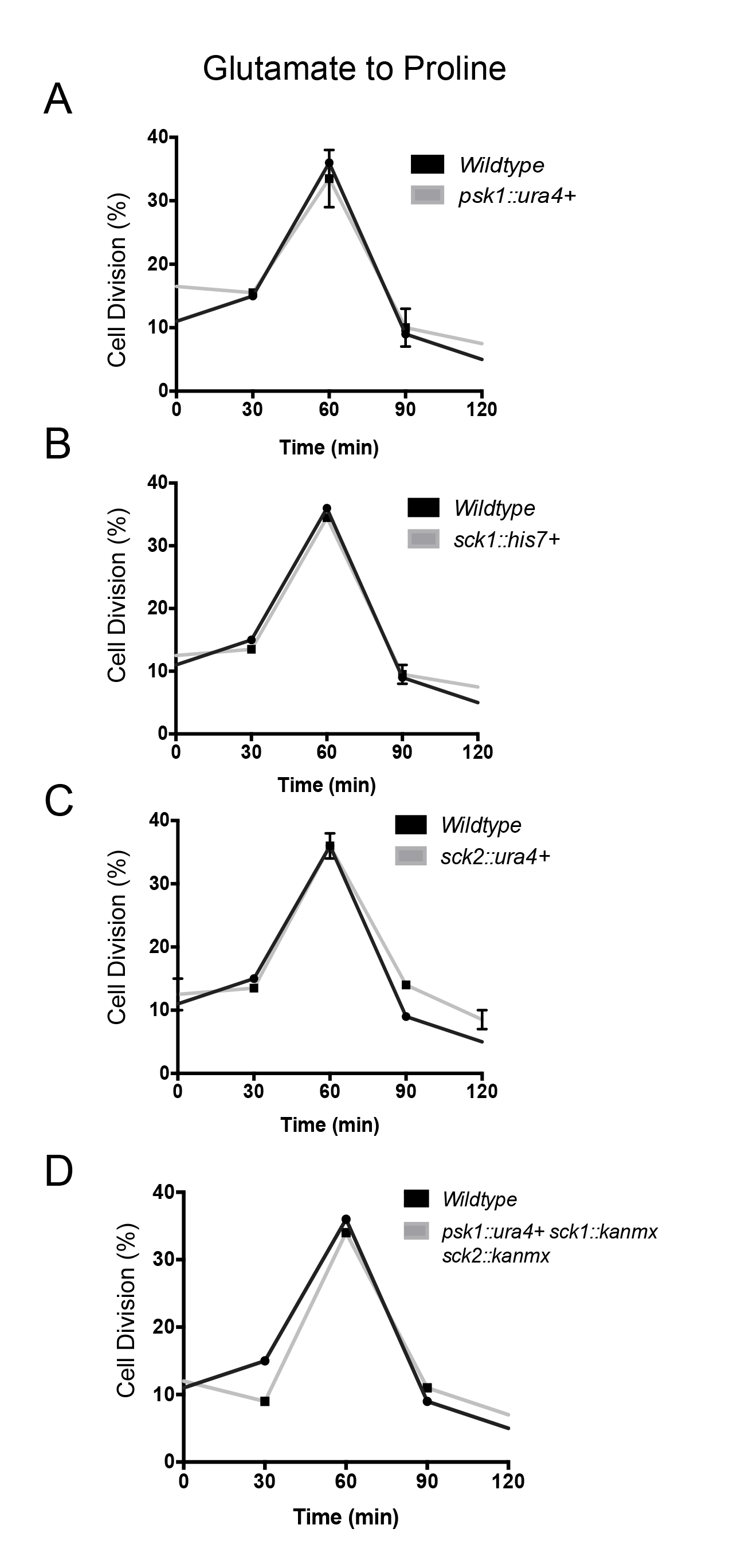

Supplement: S2 Fig — Exponentially growing cultures were shifted from media containing glutamate to proline as a nitrogen source and the numbers of dividing cells were counted at indicated time points. (TIF) [file pone.0172740.s002.tif]

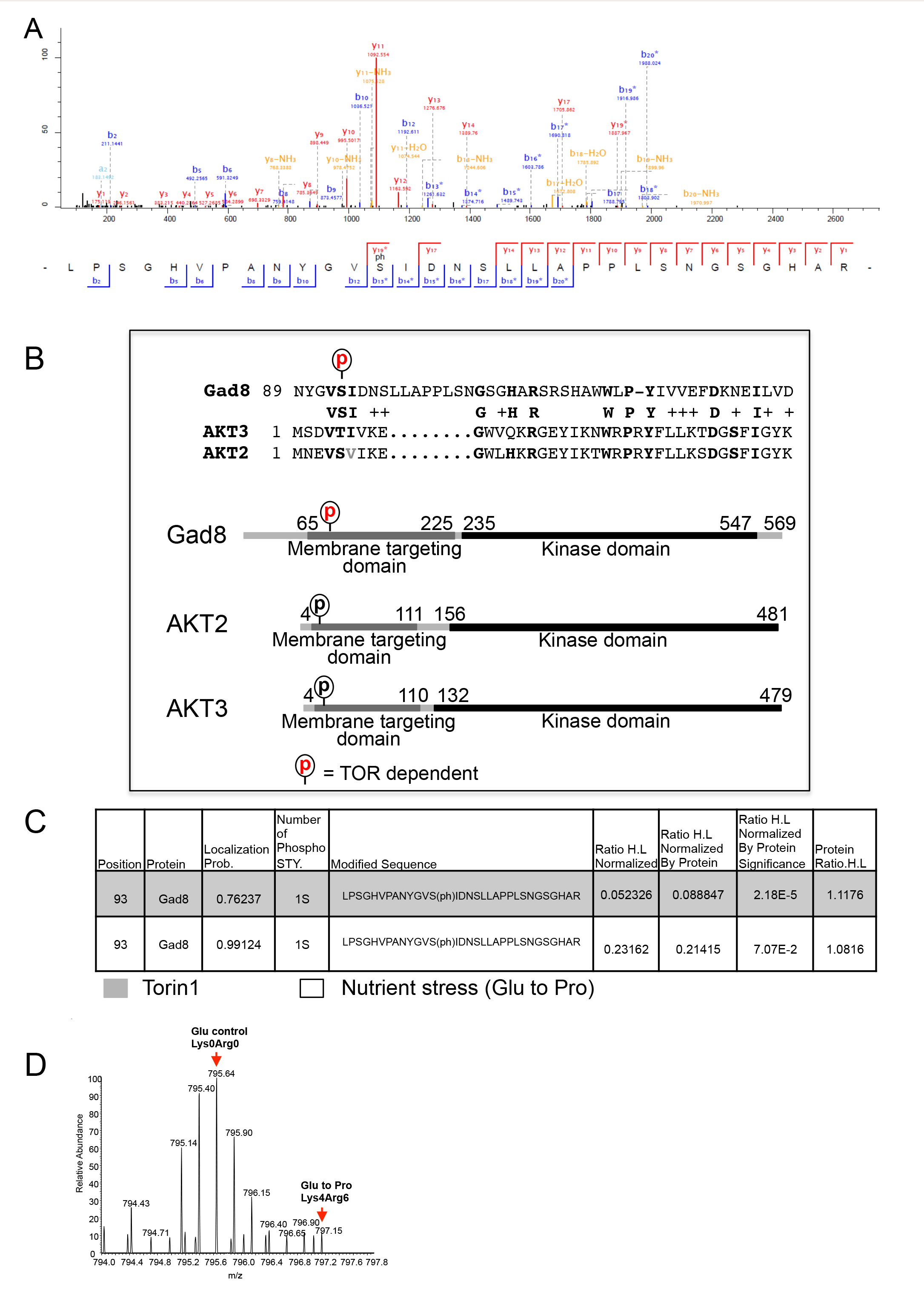

Supplement: S3 Fig — (A) The spectrum shows the fragmentation pattern of the Gad8 phosphopeptide LPSGHVPANYGVS(ph)IDNSLLAPPLSNGSGHAR indicating S93 to be phosphorylated. The mass of the parent ion is 3176.54043, the measured mass error is 0.12997 ppm. (B) The Gad8.S93 phosphorylation site may be conserved in human AKT2 and AKT3. (C,D) Gad8.S93 phosphorylation is down regulated more that 4 fold upon nitrogen stress or the addition of Torin1. D, SILAC MS spectrum showing that nitrogen stress reduces Gad8.S93 phosphorylation. (TIF) [file pone.0172740.s003.tif]
