# Supplementary material for: Ste12/Fab1 phosphatidylinositol-3-phosphate 5-kinase is required for nitrogen-regulated mitotic commitment and cell size control
Source: PLoS One. 2017 Mar 8;12(3):e0172740. doi: 10.1371/journal.pone.0172740 (PMC5342193; doi:10.1371/journal.pone.0172740)
Supplement: S1 Table — (DOCX) [file pone.0172740.s004.docx]

**S1 Table: Strains used.**

| **Strain** | **Genotype** | **Source** |
| --- | --- | --- |
| JP305 | *h^-^ leu1.32* | Lab stock |
| JP350 | *h^+^* | Lab stock |
| JP582 | *h^+^ sck1::his7 his7.36* | Lab stock |
| JP1308 | *tor1::ura4 ura4.d18* | Lab stock |
| JP1533 | *gad8::ura4 ura4.d18* | Lab stock |
| JP1731 | *h^-^ maf1pk-kanMx6* | Lab stock |
| JP1969 | *ste12.W1037^STOP^* | This study |
| JP1979 | *ste12.W1037^STOP^* *maf1pk-kanMx6* | This study |
| JP2085 | *Ste12::KanMX4* | This study  *ste12::KanMX4* deletion from Bioneer collection |
| JP2096 | *pxa1::KanMX4* | This study  *pxa1::KanMX4* deletion from Bioneer collection |
| JP2459 | *mip1.GFP:KanMX* | This study |
| JP2619 | *psk1::ura4 ura4.d18* | This study  *psk1::ura* from  M Balasubramanian |
| JP2620 | *sck2::ura4 ura4.d18* | This study  *sck2::ura* from  M Balasubramanian |
| JP2687 | *sck2::ura4 psk1::ura4 sck1::KanMX ura4.d18* | This study  *sck1::KanMX* from Bioneer collection |
| JP3124 | *ste12.W1037^STOP^* *mip1.GFP:KanMX* | This study |
| JP2848 | *Gad8.S93A* | This study |
| JP2951 | *Gad8.S93E* | This study |
